# Informing community pharmacists on COPD case-finding methods: A scoping review

**DOI:** 10.1177/17151635241284802

**Published:** 2024-10-15

**Authors:** Omowumi Idowu, Meghan Sebastianski, Janice Y. Kung, Nese Yuksel, Theresa J. Schindel, Ross T. Tsuyuki, Randy So, Tatiana Makhinova

**Affiliations:** Rexall Pharmacies ULC, Mississauga, Ontario; Faculty of Medicine and Dentistry, University of Alberta, Edmonton Clinic Health Academy, Edmonton, Alberta; Geoffrey & Robyn Sperber Health Sciences Library, University of Alberta, Edmonton Clinic Health Academy, Edmonton, Alberta; Faculty of Pharmacy and Pharmaceutical Sciences, University of Alberta, Edmonton, Alberta; Faculty of Pharmacy and Pharmaceutical Sciences, University of Alberta, Edmonton, Alberta; Department of Medicine, Faculty of Medicine and Dentistry, University of Alberta, Edmonton, Alberta; Faculty of Pharmacy and Pharmaceutical Sciences, University of Alberta, Edmonton, Alberta; Shoppers Drug Mart, Edmonton, Alberta; Faculty of Pharmacy and Pharmaceutical Sciences, University of Alberta, Edmonton, Alberta

## Abstract

**Background:**

Early detection of chronic obstructive pulmonary disease (COPD) is a strategy to address the increasing human and economic costs of this condition. This study aimed to inform pharmacists’ case-finding strategies by providing an overview of case-finding approaches by health care practitioners.

**Methods:**

A scoping review was conducted based on the Joanna Briggs Institute and the PRISMA Extension for Scoping Reviews (PRISMA ScR) guidelines. Included studies were analyzed under the following themes: population characteristics, inclusion and exclusion criteria, setting, case-finding strategies and yield, health care practitioners involved, interprofessional collaboration and the provision of preventive services. Studies were then characterized by highest yields (the weighted average of each approach expressed as a percentage of the total number of new COPD cases divided by the total number of patients screened using the same approach).

**Results:**

The screening process produced 170 eligible studies. Twenty case-finding approaches with average yields of new COPD cases ranging from 3.8% to 29% were identified. The approach with the highest yield involved the use of a questionnaire, peak flow meter and pre–post spirometry. In 14 of these approaches, the process was initiated with the use of questionnaires. In many of the studies, case-finding was targeted at high-risk and/or symptomatic patients. Characteristics of studies with the highest case-finding yields included provider education/training, patient education, active screening, multistep approaches to case-finding, provider engagement, diagnostic criteria by guidelines and engagement of other health care practitioners.

**Conclusion:**

In our scoping review of case-finding methods for patients with COPD, we found the greatest yield from pre–post spirometry following initial screening with a peak flow meter and questionnaire. Pharmacists and health researchers can use these approaches to identify high-risk patients for interventions.

**Knowledge into Practice**

- Underdiagnosis and late diagnosis of chronic obstructive pulmonary disease (COPD) is common; failure to recognize the disease and initiate timely, evidence-based care further increases the burden of COPD.
- Pharmacists can play an important role to address this gap by identifying undiagnosed patients living with COPD.
- Through this scoping review, we identified 20 case-finding approaches and provided further details of the approaches with the highest yields.
- The findings of the review provide important considerations in the planning and execution of COPD case-finding services.

## Introduction

With a global prevalence estimated at 13.1% in 2019, chronic obstructive pulmonary disease (COPD) is a leading cause of morbidity and mortality in adults.^1^ COPD is projected to be the third leading cause of death by the year 2030 due to risk factors such as gender, socioeconomic factors, an aging population and an increasing exposure to infections, outdoor/indoor air pollutants and tobacco smoke.^2-5^ The burden on the system is further compounded by underdiagnosis and late diagnosis of new patients, which lead to disease detection at advanced stages when the rates of exacerbation, attendant complications and hospitalizations are high, thus posing an enormous burden on the health system, patients and their caregivers.^6-8^ Evidence-based case-finding is a promising strategy because the identification of patients at risk improves the chances of early disease detection and treatment. Unlike screening, which commonly refers to disease testing in the general population, case-finding targets individuals who are symptomatic and/or at high risk.^9,10^

**Mise En Pratique Des Connaissances**

- Le sous-diagnostic et le diagnostic tardif de la maladie pulmonaire obstructive chronique (MPOC) sont fréquents; l’incapacité à reconnaître la maladie et à mettre en place des soins opportuns fondés sur des données probantes accroît davantage le fardeau de la MPOC.
- Les pharmaciens peuvent jouer un rôle important pour combler cette lacune en identifiant les patients atteints de la MPOC qui n’ont pas reçu de diagnostic.
- Grâce à cet examen de la portée, nous avons cerné 20 approches de recherche de cas et fourni de plus amples détails sur les approches les plus performantes.
- Les résultats de l’examen fournissent des considérations importantes pour la planification et l’exécution des services de recherche de cas de MPOC.

Aligned with the need for timely diagnoses and efficient care, COPD case-finding services have been implemented in community pharmacy settings. Pharmacists are uniquely placed to provide COPD case-finding due to their educational background and skills, accessibility to the general public and roles in direct patient care.^11^ Various approaches to case-finding such as questionnaires, microspirometry and spirometry, separately or in combination, have been used successfully by pharmacists.^12-14^

Questionnaires such as the COPD Assessment Test (CAT) and Clinical COPD Questionnaire (CCQ) are used to measure disease impact and enable health care professionals (HCPs) to effectively evaluate patients’ health status.^15^ Microspirometers are portable and inexpensive devices that measure an individual’s forced expiratory volume in 1 and 6 seconds (FEV_1_/FEV_6_), where FEV6 serves as a surrogate of forced vital capacity (FVC) in spirometry. Spirometry procedures require more skill and time and are more expensive, whereas microspirometry can help identify airflow limitation with less effort and resources.^8^

A 2015 systematic review aimed at identifying and comparing the effectiveness of COPD case-finding approaches suggested that there was no clearly optimal approach for COPD case-finding, although the evidence was restricted to primary care settings and prespecified case-finding approaches.^16^

Additional case-finding research published in the interim necessitates an updated evidence synthesis on optimal COPD case-finding strategies. We aimed to inform pharmacists’ case-finding strategies by providing an overview of case-finding approaches by HCPs, with a focus on those methods with the highest yields.

## Methods

A scoping review was conducted based on guidelines from the Joanna Briggs Institute and the PRISMA Extension for Scoping Reviews (PRISMA ScR).^17,18^ The study protocol was registered with the Open Science Framework (OSF) on January 30, 2019. This can be accessed at https://osf.io/62dx7.^19^

### Search strategy

A medical librarian (J.Y.K.) conducted comprehensive searches in Ovid MEDLINE, Ovid EMBASE, CINAHL and Web of Science on January 8, 2019, and updated on June 24, 2024. Because COPD patients are usually diagnosed at later stages, it was crucial to capture all publications related to the identification of patients at risk prior to diagnosis. This was accomplished by carefully selecting keywords and controlled vocabulary terms related to the concept surrounding early detection, screening, prescreening and preidentification. The search strategy was peer-reviewed by another health sciences librarian to ensure all possible terms were captured for the review. All searches were limited to English language. A total of 22,302 results were retrieved, and when all duplicates were removed, 10,800 unique results remained for the initial title and abstract screening. In addition to searching subscription-based databases, the research team expanded the search to grey literature and reviewed conference abstracts and relevant association websites. Hand searching of bibliographies from included studies were also conducted. The full search strategy is available in Appendix 1 in the Supplemental Materials, available online.

### Eligibility criteria

We included all primary research studies addressing COPD case-finding strategies for previously undiagnosed patients, published in the English language, with no restrictions on publication date. We excluded studies addressing screening of diseases other than COPD, comorbidity studies that did not report COPD results separately, studies aimed at validating screening tools/methods without reporting newly identified cases of COPD, studies screening for lung cancer with computed tomography scanning, studies aimed at reviewing equipment or describing screening approaches without measuring results, studies aimed at screening for α_1_-antitrypsin deficiency and studies that included diagnosed and undiagnosed patients with no subgroup analysis available for new cases. We also excluded review articles, letters, editorials, commentaries, conference abstracts and studies that were not available in full text.

### Study selection

**Figure 1:**
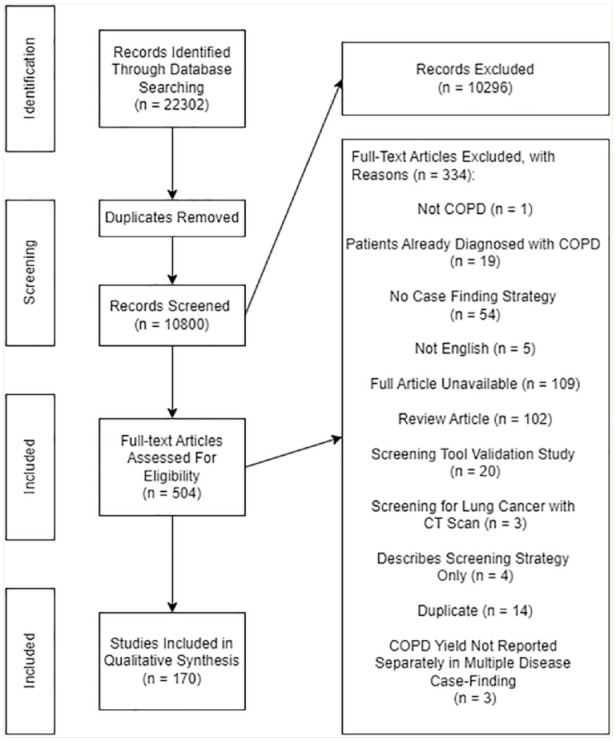
Adapted PRISMA flow diagram for the scoping review, describing the study selection process COPD, chronic obstructive pulmonary disease; CT, computed tomography.

Three researchers (O.I., S.G. and R.S.) independently reviewed the studies identified in the search against the inclusion criteria in a 2-step screening process of title and abstract only followed by full text. Disagreements were resolved by consensus or by another reviewer (T.M.). The study selection process is reported using a PRISMA diagram (Figure 1).

### Data extraction

Data extraction was an iterative process, undertaken by two authors (O.I. and R.S.) with ongoing input from team members (M.S. and T.M.). Selected review of the extracted data for accuracy was conducted (T.M.), with discrepancies discussed and resolved. Extracted data included population characteristics (mean age, sex, geographic setting), inclusion and exclusion criteria, study characteristics (setting, case-finding strategy, HCPs involved), case-finding strategies and outcomes (e.g., yield of new cases, provision of preventive services and collaborative practices among the HCPs).

### Data analysis

Approaches were classified based on the different steps involved in the case-finding process, with the differences in the case-finding approaches dependent on whether testing was conducted with a bronchodilator or whether individuals who initially underwent testing without bronchodilation had to meet preset criteria before undergoing testing with bronchodilation. Due to variation in the criteria for identifying new cases, each study’s definition of new cases was adopted. For reporting purposes, the new cases were captured as “yield”, regardless of whether the case-finding process involved confirmatory diagnosis. To analyze the yields, studies were grouped into quartiles where the study yields were sorted from smallest to largest and the quartiles calculated using Microsoft Excel.

The extracted data were analyzed using descriptive statistics and reported narratively. The yield (weighted average) of each approach was calculated as a percentage of the total number of new cases from each approach divided by the total number of patients screened using the same approach. Quartile 1 (Q1) ranged from 0.8% yield up to and including 7.3%; Quartile 2 (Q2), from 7.4% yield up to and including 13.6%; Quartile 3 (Q3), from 13.7% yield up to and including 21.0%; and Quartile 4 (Q4), from 21.1% yield up to and including 56.7%.

## Results

The search identified 10,800 unique citations; 170 articles were included (Figure 1).

Table 1 summarizes the characteristics of the included studies. Of the studies, 82% (*n* = 140) were published between 2010 and 2024. The majority (75%, *n* = 128) used a cross-sectional study design and general practice/primary care clinics (48%,*n* = 81) were the most common research setting.

**Table 1:** Characteristics of included studies (*n* = 170)

Characteristic	Description	Number of studies (%)
Year of publication	2010–2024	140 (82)
	Before 2010	30 (18)
Study design	Cross-sectional	128 (75)
	Prospective	14 (8)
	Randomized controlled trial	10 (6)
	Cohort	8 (5)
	Retrospective	4 (2)
	Longitudinal	3 (2)
	Case control	2 (1)
	Not reported	1 (1)
Geographic region	Europe	84 (49)
	Asia	45 (26)
	North America	23 (14)
	Oceania	7 (4)
	Multiregion	6 (4)
	South America	3 (2)
	Africa	1 (1)
	Not reported	1 (1)
Setting	General practice/primary care clinic	81 (48)
	Hospital	42 (25)
	Others (farm, port, community centre, outdoors, field, military training institution, home)	17 (10)
	Community pharmacy	5 (3)
	Smoking cessation centre	2 (1)
	Rehabilitation centre	2 (1)
	University	2 (1)
	Pulmonary outpatient clinic	1 (1)
	Multiple settings	1 (1)
	Hospital and pharmacy	1 (1)
	Not reported	16 (9)
Health care professionals initiating case-finding	Multidisciplinary team	60 (35)
	Physician	45 (26)
	Study personnel	13 (8)
	Nurse	10 (6)
	Respiratory therapist	7 (4)
	Pharmacist	1 (1)
	Not reported	34 (20)
Number of centres	Multicentre	74 (44)
	Single centre	53 (31)
	Not reported	43 (25)
Questionnaires used for case-finding	Standardized questionnaire	93 (55)
	Unspecified questionnaire	50 (29)
	Not applicable	25 (15)
	Not reported	2 (1)
Guideline for spirometry procedure	American Thoracic Society/European Respiratory Society	47 (28)
	American Thoracic Society	32 (19)
	GOLD guidelines	13 (8)
	European Respiratory Society	12 (7)
	Danish Respiratory Society	4 (2)
	Dutch College of General Practitioners	2 (1)
	Japanese Respiratory Society guidelines	2 (1)
	British Thoracic Society	1 (1)
	Brazilian Thoracic Association	1 (1)
	Association for Respiratory Technology & Physiology	1 (1)
	Spanish Guideline for COPD	1 (1)
	Spanish Society of Pulmonology and Thoracic Surgery	1 (1)
	Société de Pneumologie de Langue Française	1 (1)
	Not reported	50 (32)
Inclusion criteria for case-finding	Age ≥35 years	139 (81)
	Smoking history	105 (62)
	Presence of respiratory symptoms	41 (24)
	Presence of comorbidities (e.g., HIV, cardiovascular diseases)	12 (7)
	Occupational exposure	11 (6)

COPD, chronic obstructive pulmonary disease; GOLD, Global Initiative for Chronic Obstructive Lung Disease.

### Target groups for case-finding

The majority (95%, *n* = 161) of the included studies targeted individuals at high risk of COPD. The most common inclusion criteria were age ≥35 years (81%, *n* = 139) and a history of smoking (62%, *n* = 105). Other common criteria were the presence of respiratory symptoms (coughing, dyspnea and sputum production) (24%, *n* = 41), presence of comorbidities (7%, *n* = 12) and occupational exposure (6%, *n* = 11). For pharmacy-specific studies, other inclusion criteria included poorly controlled asthma, regular purchase of cough medicines/smoking cessation products and a history of antibiotic use for respiratory infection at least twice in the preceding 12 months.^12^ It is noteworthy that these criteria were not mutually exclusive; thus, in the majority of studies, more than 1 criterion for inclusion was used.

### Participant recruitment methods

Participant recruitment during routine care visits to their HCP was the most common recruitment method (26%, *n* = 45). Other strategies included random sampling of the target population (11%, *n* = 18); recruitment of patients on hospital admission (8%, *n* = 14); identification of patients through clinical/health databases or computerized medical records by using prespecified criteria (8%, *n* = 13); recruitment at large public events, conferences and congresses (5%, *n* = 9); and local advertisements (5%, *n* = 8). To raise the awareness on some of these case-finding services, studies used recruitment posters (hospitals, clinics, pharmacies or public spaces) and advertisements designed for social media, mass media and billboards.

### Health care providers and setting

HCPs initiating COPD case-finding services were most commonly physicians (general or specialists [26%, *n* = 45]), followed by study personnel (8%, *n* = 13), nurses (6%, *n* = 10), respiratory therapists (4%, *n* = 7) and pharmacists (1%, *n* = 1). In 68 studies (35%), a multidisciplinary team with a nurse, pharmacist or physician was used in initiating the case-finding process. In 20% of the studies (*n* = 34), the HCP initiating the case-finding process was either unclear or not reported. With respect to study setting, the general practice/primary care clinics were the most common (48%, *n* = 81), followed by hospital (25%, *n* = 42), others (community halls, farms, ports, outdoors, military training institutions or home) (10%, *n* = 17), community pharmacies (3%, *n* = 5), smoking cessation centres (1%, *n* = 2), pulmonary outpatient clinic (1%, *n* = 1) and multilocation settings (1%, *n* = 2). In 9% (*n* = 16) of the studies, the setting was either unclear or not reported.

### Case-finding approaches and their distribution by yield

Classification of COPD case-finding approaches was based on the different scenarios presented in the included studies. The main COPD screening tool was the questionnaire, with peak flow meter, microspirometry or spirometry used in a variety of contexts, and these were considered separately. Twenty unique approaches (Appendix 2) captured the particularities of each scenario, with most variation coming from (1) whether testing was done with a bronchodilator and (2) whether individuals who initially underwent testing without bronchodilation had to meet preset criteria before undergoing testing with bronchodilation. For example, in pre–post microspirometry/pre–post spirometry, individuals underwent testing initially without bronchodilation, immediately followed by testing with a bronchodilator. There were no set criteria for progressing to testing with bronchodilation (as with approaches #3, #6, #8, #12 and #18—see Appendix 2).

In other scenarios, individuals had to undergo an initial test without a bronchodilator (microspirometry or spirometry without bronchodilator) and then proceeded to testing with bronchodilation (bronchodilator microspirometry or bronchodilator spirometry), after they had met set criteria, for example, FEV_1_/FVC <0.7 or FEV_1_/FEV_6_ <0.7 (as with approaches #7, #10, #13, #15 and #19).

Of the 20 approaches, the most common were pre–post spirometry after initial screening with a questionnaire (approach #12: 24.7%, *n* = 42) and spirometry without bronchodilation after initial screening with a questionnaire (approach #9: 20%, *n* = 34). The least common approaches were the use of questionnaires only (approach #1: 0.6%, *n* = 1), pre–post microspirometry testing after initial screening with peak flow meter and questionnaire (approach #6: 0.6%, *n* = 1), spirometry without bronchodilation after initial screening with peak flow meter and questionnaire (approach #11: 0.6%, *n* = 1) and bronchodilator spirometry testing after screening with microspirometry (approach #16: 0.6%, *n* = 1). Of the 93 standardized questionnaires used by the included studies, the most used was the COPD Population Screener (COPD-PS) (10%, *n* = 17), followed by the COPD Assessment Test (CAT) questionnaire (10%, *n* = 17) and the Medical Research Council (MRC) questionnaire (10%, *n* = 17).

The weighted yield was an average value (range 3.8%–29%) with Q1 values ranging from 0.8% up to and including 7.3%, Q2 values ranging from 7.4% up to and including 13.6%, Q3 values ranging from 13.7% up to and including 21.0%, and Q4 values ranging from 21.1% up to and including 56.7%. Q1 has 43 studies, Q2 has 42 studies, Q3 has 42 studies and Q4 has 43 studies.

The approach with the highest yield was pre–post spirometry after initial screening with a questionnaire and peak flow meter (approach #6: 29%, *n* = 1),^20^ followed by spirometry without bronchodilation (approach #17: 28.3%, *n* = 7).^21-27^ Spirometry with bronchodilation following screening with a questionnaire, peak flow meter and spirometry without bronchodilation had the lowest yield (approach #10: 3.8%, *n* = 2).^28,29^

### Characteristics of studies with the highest yields

The studies with the highest yields, Q4 (*n* = 43), had the following shared characteristics (Table 2)^12,20,21,23-26,30-65^: case-finding approaches used had multiple steps (93%, *n* = 40/43); case-finding targeted patients at risk of COPD (93%, *n* = 40/43); the case-finding processes were HCP-led, determining steps to take in the case-finding process (88%, *n* = 38/43); patients/study participants were educated on the disease and the importance of early identification (84%, *n* = 36/43); HCPs worked together in different capacities to identify new cases (65%, *n* = 28/43); criteria for diagnosis were chosen according to clinical guidelines (63%, *n* = 27/43) and HCPs underwent training/educational activities to improve their skills, especially in conducting lung function tests (37%, *n* = 16/43).

**Table 2:** Characteristics of studies with the highest yields (Q4) (*n* = 39)

First author	Approach used^*^	Provider training/education	Patient education	Active screening	Multistep approach	Provider engagement	Diagnostic criteria by guidelines	Engagement of other health care professionals
Abbas^29^	14			•	•		•	
Alchakaki^21^	17		•	•	•	•		•
Apostolovic^30^	20			•		•	•	
Burhan^33^	13	•	•	•	•	•	•	•
Campo^20^	6		•	•	•	•	•	•
Clergue-Duval^34^	19	•	•	•	•	•	•	•
Cristescu^23^	17		•	•	•	•		
Fidalgo-Garrido^24^	17		•	•	•	•		
Geijer^35^	13	•	•	•	•	•	•	•
Geijer^36^	13	•	•	•	•	•	•	•
Górecka^38^	9		•	•	•	•		•
Hemmingsen^39^	9	•	•	•	•	•		
Hourmant^40^	2	•	•	•	•	•	•	•
Jarhyan^41^	7	•	•	•	•	•	•	•
Johnson^42^	12		•	•	•	•	•	
Kim^43^	8		•	•	•	•	•	•
Kögler^44^	12		•	•	•	•	•	•
Lambert^46^	9	•	•	•	•	•		•
Mahishale^47^	12		•	•	•	•	•	•
Marcos^48^	20		•	•	•	•	•	•
Markun^25^	17	•	•	•	•	•		•
Mets^49^	17		•	•	•	•		
Nathell^50^	19	•	•	•	•	•	•	
Ray^51^	14		•	•	•	•	•	•
Represa-Represas^52^	15	•	•	•	•	•	•	•
Riegels-Jakobsen^53^	9	•		•	•	•	•	•
Robitaille^54^	9			•	•			•
Sandelowsky^55^	19		•	•	•	•	•	•
Stratelis^56^	13	•	•	•	•	•	•	•
Sekine^57^	9		•	•	•	•		•
Steinacher^58^	18		•	•	•	•	•	
Su^59^	12			•	•		•	
Thorn^60^	8		•	•	•	•	•	
Ulrik^61^	9		•	•	•	•		
Vandevoorde^62^	9	•	•	•	•	•		
Wright^12^	2		•	•	•	•		•
Zieliñski^63^	9		•	•	•	•		•
Zhou^64^	13			•	•		•	•
Zubair^65^	12	•	•		•	•	•	•

* The 20 approaches are described in Appendix 2.

### Collaborative practices among health care professionals

Engagement of other HCPs at different stages of the case-finding process were reported by half (51%, *n* = 87) of the included studies. In 60 studies (35%), patients were referred to HCPs who could interpret or validate diagnostic results. In other studies, engagement of other HCPs was through information sharing and referral of patients to another HCP for spirometry, for smoking cessation programs, for initiation of treatment or for pulmonary rehabilitation (*n* = 51, 30%). Case-finding strategies carried out by a multidisciplinary team were seen in 29 studies (17%).

### Provision of preventive services

Smoking cessation was the only preventive service mentioned, being reported by 22% (*n* = 38) of the included studies. Smoking cessation was offered to patients in 2 instances, following confirmed diagnosis or risk assessment (high-risk category). Smoking cessation programs were conducted by either the HCP who initiated the case-finding or other HCPs to whom patients were referred. In the studies where pharmacists initiated the case-finding process, they also provided smoking cessation services to eligible patients.

### Studies conducted in the community pharmacy setting

Five studies were conducted in the community pharmacy setting.^12-14,66,67^ Their yields were 1.9% (Q1), 3.2% (Q1), 9% (Q2), 9.6% (Q2) and 56.7% (Q4). Of these 5 studies, 2 studies used approach #2 (questionnaire followed by microspirometry without bronchodilation), 2 studies used approach #9 (questionnaire followed by spirometry without bronchodilation) and 1 study used approach #4 (microspirometry without bronchodilation followed by spirometry without bronchodilation after screening with a questionnaire). In all the studies, pharmacists collaborated with general physicians by referring patients with suspected COPD for further clinical evaluation, conventional spirometry and initiation of treatment. For the study with the greatest yield (56.7%), pharmacists followed approach #2 (questionnaire followed by microspirometry without bronchodilation).^12^ In another study, pharmacists collaborated with pulmonologists in addition to general physicians.^66^ The pulmonologists provided training to pharmacists on lung function testing and validated the tests conducted by the pharmacists.

Where applicable, information sharing initiated by the pharmacists to communicate with the physicians was facilitated by a referral form (screening, brief patient history, reason for referral, etc.), and a questionnaire/feedback form was initiated by physicians to communicate with pharmacists (diagnosis, therapeutic action and recommendations for the pharmacist to follow up with the patient). It is important to note that the referral and questionnaires/feedback forms were used solely for study purposes. In 4 of these studies, pharmacists underwent training programs on conducting screening and identifying patients at risk of COPD, conducting spirometry tests, analyzing and interpreting spirometry results and providing ongoing support to diagnosed patients.

## Discussion

This study aimed to inform pharmacists’ case-finding strategies by providing an overview of case-finding approaches by HCPs and characterizing studies with the highest yields. Using a detailed method of recording the components of the case-finding process, we identified 20 case-finding approaches. The studies were further classified based on the yield, and several important commonalities were identified from the studies with high yield. The following characteristics were identified: targeted screening, multistep approaches to case-finding, diagnostic criteria by guidelines, provider training, patient education, provider engagement, collaboration with other HCPs and provision of preventative services. Furthermore, we identified an approach (approach #2) that has previously been used in a community pharmacy setting and has had success in case-finding.^12^ The study findings may enhance COPD case-finding provided by community pharmacists, whose accessibility to the public represents an excellent opportunity to identify patients with COPD.

On reviewing the characteristics of participants recruited in the included studies, we noted that case-finding approaches were primarily targeted toward symptomatic (presence of respiratory symptoms) individuals and/or those at high risk of COPD (e.g., patients who currently smoke). This is consistent with recommendations by the National Institute for Health and Care Excellence (NICE) guidelines, the Global Initiative for Chronic Obstructive Lung Disease (GOLD) guidelines and the United States Preventive Services task force that case-finding is more effective in the symptomatic population.^8,68,69^ In 2 of the included studies, participants were healthy/asymptomatic with yields of 12.6% and 50.5%.^29,70^ In addition to respiratory symptoms and the commonly known risk factors, other target groups may include patients with HIV, elderly patients with complaints of dyspnea or exercise intolerance or patients with cardiovascular diseases.^32,46,47,58,71^ In the studies with the highest yields except one, case-finding services were executed by actively targeting symptomatic/high-risk groups.^29^ This reflects an alignment with the guidelines’ recommendation of conducting case-finding in symptomatic individuals.

On examination of the approaches, the most deployed case-finding approaches (#9 and #12) were not those with the highest weighted average yield. The approaches with the highest weighted average yield were approaches #6, #17 and #20 (29%, 28% and 26%, respectively), which consisted of 12 studies. In most of these 12 studies (*n* = 10, 83%), criteria for diagnosis followed those recommended by GOLD (postbronchodilator spirometry FEV_1_/FVC <70%). Adherence to the COPD diagnostic criteria recommended by the guidelines is essential in reducing misdiagnosis, which may lead to an overestimation of actual cases and patients receiving incorrect treatment. Overestimation of actual cases may have been responsible for the differences in the case-finding yields between the studies in which the GOLD diagnostic criteria were not used to identify new cases (*n* = 42 of the total 170 studies, 25%) and in those studies where new cases were confirmed following the GOLD guidelines (*n* = 128, 75%). Overestimation of cases may cause psychological challenges to the patient and a waste of health care resources by the system. Thus, beyond the potential number of new cases an approach may identify, it is important to ensure that such an approach leads to correct and accurate disease diagnosis.

Provider education/training was an important part of the studies with the highest yields. Training empowers HCPs to accurately identify new cases.^72,73^ Training may also help to reduce COPD misdiagnosis, which is commonly associated with inadequate skill with physicians in general practice.^73^ In the studies where providers were educated/trained, the training programs were mostly targeted at enhancing/ensuring the capability of the providers to conduct the case-finding tests appropriately, specifically microspirometry and spirometry. In some cases, the providers were also taught how to interpret test results. Among the studies that involved an educational/training component, some were intervention studies whereas others were observational.

In the studies with the highest yields, patients were educated or provided with additional information about their symptoms, risk factors and the disease itself to increase awareness.^38,51,53^ The absence of this information is a challenge to COPD diagnosis.^74^ In COPD case-finding, educating symptomatic individuals or those at risk of COPD may increase their awareness about their health and the disease, thus potentially influencing their willingness to undergo and complete case-finding services as well as to seek out services to help reduce their risk, such as smoking cessation. This is consistent with the knowledge that education is a critical factor in patients’ participation in decision-making.^75^

Collaboration with other HCPs affords the benefit of leveraging each other’s knowledge, resources, skill or capability. Specifically for the studies conducted in the community pharmacy settings, it was identified that pharmacists referred patients to other HCPs such as general physicians and pulmonologists for confirmatory diagnosis, for the initiation of smoking cessation programs, for initiation of treatment/pulmonary rehabilitation and for educational services.^12-14,30,31^ Focusing on the studies in the pharmacy setting, it is evident that collaboration with other HCPs is key to successful COPD case-finding by pharmacists. It is also important that communication lines between the pharmacists and the collaborating HCPs are open, and a feasible information sharing strategy and tools are mapped out and followed, to ensure that each HCP has the relevant patient information needed for clinical decisions and also to ensure patients are not lost to follow-up within the system.

Case-finding was commonly practice-managed (provider-led), thereby supporting more patients to complete the case-finding process. This is more effective than patient-managed case-finding scenarios, where patients self-administer the screening questionnaires and reach out to HCPs for next steps, if they are interested.^76^

Of the preventive services of interest to us, none of the included studies reported the administration of vaccines to newly identified COPD cases and only a few reported the initiation/recommendation of smoking cessation programs. Although this may have been beyond the scope of the included studies, we suggest that in practice, these services should be consistently available to patients living with COPD because smoking cessation is the most effective treatment in altering the progression of COPD and vaccination reduces the susceptibility of COPD patients to recurrent respiratory infections.^77,78^

The majority of included studies were published between 2010 and 2024, which reflects the impact of the more recent evidence that the most rapid decline in lung function occurs at early stages of disease.^79,80^ This evidence is contrary to the prior notion that the rate of lung function decline is directly proportional to the time progression of COPD.^81^ Thus, an awareness that the most rapid lung function decline has early onset may have led to the increased research on early COPD identification. Furthermore, due to the evolution of HCP roles over the years, more involvement of other HCPs apart from physicians (pharmacists included) in identifying individuals who may be at risk of COPD based on the presence of symptoms and/or risk factors has been observed.

### Limitations

This scoping review has a few limitations. Only studies published in English were included, which may have led to the exclusion of relevant studies in other languages. Not all the studies used the clinical guideline recommendations as their diagnostic criteria, which may have led to an inflated yield of new cases. Inadequate information by some studies also led to a high number of unreported/unsure cases in some of our results. In some studies, participants were lost to follow-up, and in some cases of interprofessional collaboration, determining the number of at-risk individuals who were appropriately diagnosed was problematic due to poor follow-up/continuity of care and sharing of information. Further, in calculating the weighted average yields for the different approaches, the influence of the number of studies each approach had was not considered. The yields may have been influenced by other factors, such as study design, recruitment strategy, study setting and HCPs involved, and are not solely dependent on the case-finding approaches. Last, it is acknowledged that our findings and considerations for pharmacy practice may have been affected by the methodological limitations/risk of bias of the individual studies, which were not analyzed. Only 5 of the 170 included studies were conducted at community pharmacies. Thus, an assumption is that the best approaches could be considered and used by pharmacists. Given the importance of COPD and poor outcomes, we recommend the following approach based upon our scoping review: screening with a questionnaire, followed by peak flow meter, and then pre–post spirometry (approach #6), as this demonstrated the highest average yield.

## Conclusion

This review identified 20 case-finding approaches used by different HCPs across various practice settings. The approach with the highest yield consisted of pre–post spirometry following initial screening with a peak flow meter and questionnaire. This review also identified some considerations that may be useful in the planning of case-finding services by pharmacists. Collaborative practices involved case-finding in multidisciplinary teams or referral of patients from one HCP to another during the case-finding process. Pharmacists are well-positioned to ease the burden of COPD on the system and patients through identification of undiagnosed COPD cases and the delivery of patient-centred COPD management services.

## Supplemental Material

sj-pdf-1-cph-10.1177_17151635241284802 – Supplemental material for Informing community pharmacists on COPD case-finding methods: A scoping review Supplemental material, sj-pdf-1-cph-10.1177_17151635241284802 for Informing community pharmacists on COPD case-finding methods: A scoping review by Omowumi Idowu, Meghan Sebastianski, Janice Y. Kung, Nese Yuksel, Theresa J. Schindel, Ross T. Tsuyuki, Randy So and Tatiana Makhinova in Canadian Pharmacists Journal / Revue des Pharmaciens du Canada
