# Supplementary material for: Informing community pharmacists on COPD case-finding methods: A scoping review
Source: Can Pharm J (Ott). 2024 Oct 15;157(6):290–303. doi: 10.1177/17151635241284802 (PMC11556641; doi:10.1177/17151635241284802)
Supplement: sj-pdf-1-cph-10.1177_17151635241284802 – Supplemental material for Informing community pharmacists on COPD case-finding methods: A scoping review [file sj-pdf-1-cph-10.1177_17151635241284802.pdf]

APPENDIX 1. Search strategy methods

Database	Search strategy
**MEDLINE**<br><br>**Ovid MEDLINE(R) ALL** 1946 to June 24, 2024	1. exp Pulmonary Disease, Chronic Obstructive/ or Lung Diseases, Obstructive/<br>2. (chronic obstructive or emphysema or COPD or COAD or AECB or airflow limitation*).mp.<br>3. (chronic adj3 bronchiti*).mp.<br>4. #1 or #2 or #3<br>5. (preidentif* or pre-identif* or predetect* or pre-detect*).mp.<br>6. (((early or earlier or pre-symptom* or presymptom*) adj3 (identif* or detect*)) and (screen* or surveill*)).ab.<br>7. ((early or earlier or pre-symptom* or presymptom*) adj3 (screen* or surveill*)).ab.<br>8. screen*.ti. or mass screening/<br>9. (predict* and (COPD or COAD or AECB or chronic obstructive or (chronic adj3 bronchiti*) or emphysema)).ti,kf.<br>10. ((screen* or predict*) adj3 (COPD or COAD or AECB or chronic obstruct* or (chronic adj3 bronchiti*) or emphysema)).ti,ab,kf<br>11. case finding*.mp.<br>12. or/#5–11<br>13. #4 and #12<br>14. limit #13 to english language
**Embase**<br><br>**Ovid Embase** 1974 to June 24, 2024	1. exp chronic obstructive lung disease/<br>2. (chronic obstructive or emphysema or COPD or COAD or AECB or airflow limitation*).mp.<br>3. (chronic adj3 bronchiti*).mp.<br>4. #1 or# 2 or #3<br>5. (preidentif* or pre-identif* or predetect* or pre-detect*).mp<br>6. (((early or earlier or pre-symptom* or presymptom*) adj3 (identif* or detect*)) and (screen* or surveill*)).ab.<br>7. ((early or earlier or pre-symptom* or presymptom*) adj3 (screen* or surveill*)).ab.<br>8. screen*.ti. or mass screening/<br>9. (predict* and (COPD or COAD or AECB or chronic obstructive or (chronic adj3 bronchiti*) or emphysema)).ti,kw.<br>10. ((screen* or predict*) adj3 (COPD or COAD or AECB or chronic obstruct* or (chronic adj3 bronchiti*) or emphysema)).ti,ab,kw.<br>11. case finding*.mp.

	12. or/#5–11<br>13. #4 and #12<br>14. limit #13 to english language
**CINAHL**	1. (MH "Pulmonary Disease, Chronic Obstructive+")<br>2. (MH "Lung Diseases, Obstructive+")<br>3. "chronic obstructive" or emphysema or COPD or COAD or AECB or "airflow limitation*"<br>4. chronic N3 bronchiti*<br>5. #1 OR #2 OR #3 OR #4<br>6. preidentif* or "pre-identif*" or predetect* or "pre-detect*"<br>7. AB (((early or earlier or "pre-symptom*" or presymptom*) N3 (identif* or detect*)) and (screen* or surveill*))<br>8. ((early or earlier or "pre-symptom*" or presymptom*) N3 (screen* or surveill*))<br>9. TI screen*<br>10. (predict* and (COPD or COAD or AECB or "chronic obstructive" or (chronic N3 bronchiti*) or emphysema))<br>11. ((screen* or predict*) N3 (COPD or COAD or AECB or "chronic obstruct*" or (chronic N3 bronchiti*) or emphysema))<br>12. "case finding*"<br>13. #6 OR #7 OR #8 OR #9 OR #10 OR #11 OR #12<br>14. #5 AND #13 (limiters: English language)
**Web of Science All Databases**	1. TI=("Chronic Obstructive Pulmonary Disease" or emphysema* or copd or coad or aecb or "airflow limitation*")<br>2. TI=(chronic NEAR/3 bronchiti*)<br>3. #1 or #2<br>4. TS=((early or earlier or "pre-symptom*" or presymptom*) NEAR/3 (screen* or surveill*))<br>5. TI=screen*<br>6. TI=(predict* and (COPD or COAD or AECB or chronic obstructive or (chronic NEAR/3 bronchiti*) or emphysema))<br>7. TS=((screen* or predict*) NEAR/3 (COPD or COAD or AECB or "chronic obstruct*" or (chronic NEAR/3 bronchiti*) or emphysema))<br>8. TI=("case finding*")<br>9. #4 or #5 or #6 or #7 or #8<br>10. #3 and #9<br>11. Refined by LANGUAGES: (ENGLISH)

APPENDIX 2. Overview of case-finding approaches and their distribution by yield of new cases (n = 170)

Approach*	Reference	Step 1	Step 2	Step 3	Step 4	No of studies, (%)	0.8<Q1≤ 7.3	7.3< Q2≤ 13.6	13.6< Q3≤ 21.0	21.0< Q4≤ 56.7	Weighted Average yield (%)**
1	Seamark et al.[1]	Questionnaire				1 (0.6%)	0	1	0	0	8.7
2	Fathima et al.[2], **Halpin et al.[3], Hourmanta et al.[4],** Richard et al.[5], Sui et al.[6], **Wright et al.[7],** Yawn et al.[8]	Questionnaire	Microspirometry without bronchodilation			7 (4.1%)	1	2	1	3	19.4
3	Estes et al.[9], Van der Velden et al.[10]	Questionnaire	Pre–post microspirometry			2 (1.2%)	1	1	0	0	8.7
4	Allan et al.[11], Crooks et al.[12], Nelson et al.[13], Wada et al.[14], Yau et al.[15]	Questionnaire	Microspirometry without bronchodilation	Spirometry without bronchodilation		5 (2.9%)	2	2	1	0	9.9
5	Ching et al.[16], Kobayashi et al.[17]	Questionnaire	Microspirometry without bronchodilation	Spirometry with bronchodilation		2 (1.3%)	2	0	0	0	4.6
6	**Campo et al.[18]**	Questionnaire	Peak flow meter	Pre–post spirometry		1 (0.6%)	0	0	0	1	29
7	**Jarhyan et al.[19],** Jouneau et al.[20]	Questionnaire	Microspirometry without bronchodilation	Spirometry without bronchodilation	Spirometry with bronchodil ation	2 (1.3%)	1	0	0	1	15.3

8	Kaufmann et al.[21],<br>**Kim et al.[22],**<br>Liang et al.[23]<br>**Thorn et al.[24]**	Questionnaire	Microspirometry without bronchodilation	Pre–post spirometry		4 (2.4%)	1	0	1	2	17.5
9	AH et al.[25],<br>**Baker et al.[26],**<br>Balata et al.[27],<br>Bednarek.[28],<br>Bucchieri et al.[29],<br>Buffels et al.[30],<br>Castillo et al.[31],<br>DeJong et al.[32],<br>Dirven et al.[33],<br>Fuller et al.[34],<br>**Gorecka et al.[35],**<br>Haroon et al.[36],<br>**Hemmingsen et al.[37],**<br>Hepper et al.[38],<br>Iyer et al. [39],<br>Jordan et al.[40],<br>Korczynski et al.[41],<br>**Lambert et al.[42],**<br>Lyngso et al.[43],<br>Maio et al.[44],<br>Manzar et al.[45],<br>Oike et al.[46],<br>Qazi et al.[47],<br>**Riegels-Jakobsen et al.[48],**<br>**Robitaille et al.[49],**<br>Sekine et al.[50],<br>**Sekine et al.[51],**	Questionnaire	Spirometry without bronchodilation			34 (20%)	9	6	9	10	16.9

	Tabori et al.[52], **Ulrik et al.[53],** Schayck et al.[54], **Vandevoorde et al.[55],** Wang et al.[56], Wisnivesky et al.[57], **Zielinski and Rokach et al.[58]**										
10	Shirley et al.[59], Soriano et al.[60]	Questionnaire	Peak flow meter	Spirometry without bronchodilation	Spirometry with bronchodilation	2 (1.1%)	2	0	0	0	3.8
11	Mahboub et al.[61]	Questionnaire	Peak flow meter	Spirometry without bronchodilation		1 (0.6%)	0	1	0	0	12.9
12	Aaron et al.[62], Al Ghobain et al.[63], Alhabeeb et al.[64], Andreeva et al.[65], Bahtouee et al.[66], **Bertens et al.[67],** Bui et al.[68], Bunker et al.[69], Chapron et al.[70], Dirven et al.[71], Grzetic-Romcevic et al.[72], Hanibuchi et al.[73], Hvidsten et al.[74],	Questionnaire	Pre–post spirometry			42 (24.7%)	10	12	14	6	14.1

	Jithoo et al.[75], **Johnson et al.[76],** Jordan et al.[77], KalagoudaMahishale et al.[78], Kart et al.[79], Kjeldgaard et al.[80], **Kögler et al.[81]**, Laniado-Laborin et al.[82], Lewis-Burke et al.[83], Lopez Varela et al.[84], **Mahishale et al.[85],** Midtgarden et al.[86], Nascimento et al.[87], Pagano et al.[88], Preteroti et al.[89], Sang et al.[90], Sansores et al.[91], Schirnhofer et al.[92], Siddharthan et al.[93], Sinha et al.[94], Skucha et al.[95], Stav and Raz[96], **Su et al.[97],** Tang et al.[98], Van Mourik et al.[99],										

	Van Mourik et al.[100], Yang et al.[101], Zachariades et al.[102], **Zubair et al.[103]**										
13	Agarwal et al.[104], Alam et al.[105], Bednarek et al.[106], **Burhan et al.[107],** Demirci et al.[108], Dickinson et al.[109], **Geijer et al.[110], Geijer et al.[111],** Gu et al.[112], Hadstad et al.[113], Hill et al.[114], Jan et al.[115], Khassawneh et al.[116], Konstantikaki et al.[117], Kotaki et al.[118]. Laraqui et al.[119], Llordes et al.[120], **Løkke et al.[121],** Lu et al.[122], Martinez et al.[123], Mycroft et al.[124], Ozemek et al.[125],	Questionnaire	Spirometry without bronchodilation	Spirometry with bronchodilation		30 (17.6%)	9	8	7	6	13.4

	Quiros-Roldan et al.[126], Spyratos et al.[127], Stafyla et al.[128], Stanley et al.[129], **Stratelis et al.[130],** Undrunas et al.[131], Zhao et al.[132], **Zhou et al.[133]**										
14	**Abbas et al.[134],** Capozzolo et al.[135], Dirven et al.[136], Hanibuchi et al.[137], Lee et al.[138], **Ray et al.[139],** Toljamo et al.[140], Tsukuya et al.[141], Yang et al.[142]	Questionnaire	Spirometry with bronchodilation			9 (5.3%)	4	3	0	2	14.3
15	Kjeldgaard et al.[143], **Represas-Represas et al.[144]**	No bronchodilation microspirometry	Spirometry without bronchodilation	Spirometry with bronchodilation		2 (1.2%)	0	0	1	1	23.9
16	Labor et al.[145]	Microspirometry without bronchodilation	Spirometry with bronchodilation			1 (0.6%)	0	0	1	0	18.9
17	**Alchakaki et al.[146],** Catlett & Kidera[147] ,	Spirometry without bronchodilation				7 (4.1%)	1	0	1	5	28.3

	**Cristescu et al.[148], Fidalgo-Garrido et al.[149], Markun et al.[150], Mets et al.[151],** Takahashi et al.[152]										
18	Boschetto et al.[153], Brenner et al.[154], Hamers et al.[155], Mosharraf-Hossain et al.[156], Queiroz et al.[157], **Steinacher et al.[158]**	Pre–post spirometry				6 (3.5%)	0	3	2	1	15.8
19	Clergue-Duval et al.[159], Freeman et al.[160], Mooe & Stenfors[161], **Nathell et al.[162],** Ronaldson et al.[163], **Sandelowsky et al.[164],** Tupper et al.[165], **Vrijhoef et al.[166]**	Spirometry without bronchodilation	Spirometry with bronchodilation			8 (4.7%)	0	2	3	3	18.5
20	**Apostolovic et al.[167],** Barthwal & Singh[168], **Marcos et al.[169],**	Spirometry with bronchodilation				4 (2.4%)	0	1	1	2	26.3

	Tinkelman et al.[170]										

*Bolded approaches with the highest yield (Q4).
**Weighted average yield calculated as a % of the (division of the total number of the new cases in each approach / total number of patients screened using the same approach).

References

1. Seamark DA, Williams S, Timon S, Ward A, Ward D, Seamark C, et al. Home or surgery based screening for chronic obstructive pulmonary disease (COPD)? Prim Care Respir J. 2001 Jun;10(2):30–3.
2. Fathima M, Saini B, Foster J, Armour C. Community pharmacy-based case finding for COPD in urban and rural settings is feasible and effective. Int J Chron Obstruct Pulmon Dis. 2017 Sep;Volume 12:2753–61.
3. Halpin D, Holmes S, Calvert J, McInerney D. Case finding for chronic obstructive pulmonary disease in people attending long-term condition clinics in primary care. Chron Respir Dis. 2016 Nov;13(4):337–43.
4. Hourmant B, Gobert CG, Plumet R, Lott MC, Zabbé C, Tromeur C, et al. Screening for COPD in primary care, involving dentists, pharmacists, physiotherapists, nurses and general practitioners (the UNANIME pilot study). Respir Med Res. 2021 Nov 1;80:100853.
5. Richard P, Gilles H, Alavi Z, Christine L, Maryline LB, Ronan G, et al. Screening for Chronic Obstructive Pulmonary Disease in Smoking Cessation Clinic in France. Addict Health. 2016;8(1):1–8.
6. Sui CF, Ming LC, Neoh CF, Ibrahim B. VitalQPlus: a potential screening tool for early diagnosis of COPD. Int J Chron Obstruct Pulmon Dis. 2015 Aug 11;10:1613–22.
7. Wright D, Twigg M, Thornley T. Chronic obstructive pulmonary disease case finding by community pharmacists: a potential cost-effective public health intervention. Int J Pharm Pract. 2015;23(1):83–5.
8. Yawn BP, Duvall K, Peabody J, Albers F, Iqbal A, Paden H, et al. The Impact of Screening Tools on Diagnosis of Chronic Obstructive Pulmonary Disease in Primary Care. Am J Prev Med. 2014 Nov;47(5):563–75.
9. Estes TS, Short N, Bowser D, Boyle A. An evidence-based quality improvement perspective for a chronic obstructive pulmonary disease case-finding program. Chron Respir Dis. 2014 Aug;11(3):131–8.
10. Allan H, Diamandis S, Saini DB, Marshall MD, Guy D, Peterson-Clark DG. A COLLABORATIVE SCREENING, REFERRAL AND MANAGEMENT PROCESS TO IMPROVE HEALTH OUTCOMES IN CHRONIC OBSTRUCTIVE PULMONARY DISEASE (COPD). FINAL Rep. :36.
11. van der Velden RMJ, Hereijgers MJM, Arman N, van Middendorp N, Franssen FME, Gawalko M, et al. Implementation of a screening and management pathway for chronic obstructive pulmonary disease in patients with atrial fibrillation. Europace. 2023 Jul 4;25(7):euad193.
12. Crooks MG, Thompson JL, Cummings H, Watkins K, Jackson N, Platten S, et al. Hidden morbidity: The results of a collaborative community chronic obstructive pulmonary disease screening initiative. Clin Respir J. 2019 Jan;13(1):43–9.
13. Nelson SB, LaVange LM, Nie Y, Walsh JW, Enright PL, Martinez FJ, et al. Questionnaires and Pocket Spirometers Provide an Alternative Approach for COPD Screening in the General Population. Chest. 2012 Aug;142(2):358–66.

14. Wada H, Nakano Y, Nagao T, Osawa M, Yamada H, Sakaguchi C, et al. Detection and prevalence of chronic obstructive pulmonary disease in a cardiovascular clinic: Evaluation using a hand held FEV1/FEV6 meter and questionnaire: Detection and prevalence of COPD. Respirology. 2010 Nov;15(8):1252–8.
15. Department of Family Medicine, Penang Medical College, 4, Jalan Sepoy Lines, 10450 Pulau Pinang, Malaysia, Kooi Yau C, Rahim FF, Department of Public Health, Penang Medical College, 4, Jalan Sepoy Lines, 10450 Pulau Pinang, Malaysia, Jiunn Sheng C, Department of Family Medicine, Penang Medical College, 4, Jalan Sepoy Lines, 10450 Pulau Pinang, Malaysia, et al. Assessing Airflow Limitation among Smokers in a Primary Care Setting. Malays J Med Sci. 2018;25(3):78–87.
16. Ching SM, Pang YK, Price D, Cheong AT, Lee PY, Irmi I, et al. Detection of airflow limitation using a handheld spirometer in a primary care setting. Respirol Carlton Vic. 2014 Jul;19(5):689–93.
17. Kobayashi S, Hanagama M, Yanai M, for the Ishinomaki COPD Network (ICON) Investigators. Early Detection of Chronic Obstructive Pulmonary Disease in Primary Care. Intern Med. 2017;56(23):3153–8.
18. Campo G, Pavasini R, Barbetta C, Maietti E, Mascetti S, Biscaglia S, et al. Predischarge screening for chronic obstructive pulmonary disease in patients with acute coronary syndrome and smoking history. Int J Cardiol. 2016 Nov;222:806–12.
19. Jarhyan P, Hutchinson A, Khatkar R, Kondal D, Botti M, Prabhakaran D, et al. Diagnostic Accuracy of a Two-Stage Sequential Screening Strategy Implemented by Community Health Workers (CHWs) to Identify Individuals with COPD in Rural India. Int J Chron Obstruct Pulmon Dis. 2021 Apr 29;16:1183–92.
20. Jouneau S, Boché A, Brinchault G, Fekete K, Guillot S, Bayat S, et al. On-site screening of farming-induced chronic obstructive pulmonary disease with the use of an electronic mini-spirometer: results of a pilot study in Brittany, France. Int Arch Occup Environ Health. 2012 Aug;85(6):623–30.
21. Kaufmann M, Hartl S, Geyer K, Breyer MK, Burghuber OC. Measuring FEV6 for Detecting Early Airway Obstruction in the Primary Care Setting. Respiration. 2009;78(2):161–7.
22. Kim JK, Lee CM, Park JY, Kim JH, Park S hoon, Jang SH, et al. Active case finding strategy for chronic obstructive pulmonary disease with handheld spirometry: Medicine (Baltimore). 2016 Dec;95(50):e5683.
23. Liang J, Abramson MJ, Zwar NA, Russell GM, Holland AE, Bonevski B, et al. Diagnosing COPD and supporting smoking cessation in general practice: evidence–practice gaps. Med J Aust. 2018;208(1):29–34.
24. Thorn J, Tilling B, Lisspers K, Jörgensen L, Stenling A, Stratelis G. Improved prediction of COPD in at-risk patients using lung function pre-screening in primary care: a real-life study and cost-effectiveness analysis. Prim Care Respir J J Gen Pract Airw Group. 2012 Jun;21(2):159–66.
25. AH F, Ban Yu-Lin A, M N, Izuanuddin A, H T. Defining the Prevalence and Predictors of Restrictive and Obstructive Airway Pattern in a Non-Selected Malaysian Population. Med Health. 2020 Dec 31;15(2):140–52.
26. Baker TR, Oscherwitz M, Corlin R, Jarboe T, Teisch J, Nichaman MZ. Screening & Obstructive Treatment Program Pulmonary Disease for Mild Chronic. JAMA. 1970 Nov 23;214(8):1448–55.
27. Balata H, Harvey J, Barber PV, Colligan D, Duerden R, Elton P, et al. Spirometry performed as part of the Manchester community-based lung cancer screening programme detects a high prevalence of airflow obstruction in individuals without a prior diagnosis of COPD. Thorax. 2020 Aug 1;75(8):655–60.

28. Zieliñski J, Bednarek M. Early Detection of COPD in a High-Risk Population Using Spirometric Screening. Chest. 2001 Mar;119(3):731–6.
29. Buffels J, Degryse J, Heyrman J, Decramer M. Office Spirometry Significantly Improves Early Detection of COPD in General Practice. Chest. 2004 Apr;125(4):1394–9.
30. Bucchieri S, Alfano P, Audino P, Fazio G, Marcantonio S, Cuttitta G. Airway Obstruction in Primary Care Patients: Need for Implementing Spirometry Use. Diagnostics (Basel). 2022 Nov 3;12(11):2680.
31. Castillo D, Burgos F, Guayta R, Giner J, Lozano P, Estrada M, et al. Airflow obstruction case finding in community-pharmacies: A novel strategy to reduce COPD underdiagnosis. Respir Med. 2015 Apr;109(4):475–82.
32. DeJONG SR, Veltman RH. The Effectiveness of a CNS-led Community-based COPD Screening and Intervention Program. Clin Nurse Spec. 2004 Apr;18(2):72.
33. Dirven JA, Tange HJ, Muris JW, van Haaren KM, Vink G, van Schayck OC. Early detection of COPD in general practice: patient or practice managed? A randomised controlled trial of two strategies in different socioeconomic environments. Prim Care Respir J J Gen Pract Airw Group. 2013 Sep;22(3):331–7.
34. Fuller L, Conrad WF, Heaton PC, Panos R, Eschenbacher W, Frede SM. Pharmacist-managed chronic obstructive pulmonary disease screening in a community setting. J Am Pharm Assoc. 2012 Sep;52(5):e59–66.
35. Górecka D, Bednarek M, Nowiński A, Puścińska E, Goljan-Geremek A, Zieliński J. Diagnosis of airflow limitation combined with smoking cessation advice increases stop-smoking rate. Chest. 2003 Jun;123(6):1916–23.
36. Haroon S, Adab P, Griffin C, Jordan R. Case finding for chronic obstructive pulmonary disease in primary care: a pilot randomised controlled trial. Br J Gen Pract. 2013 Jan 1;63(606):e55–62.
37. Hemmingsen UB, Stycke M, Dollerup J, Poulsen PB. Guideline-Based Early Detection of Chronic Obstructive Pulmonary Disease in Eight Danish Municipalities: The TOP-KOM Study. Pulm Med. 2017;2017:1–5.
38. Hepper NGG, Drage CW, Davies SF, Rupp WM, LaMothe J, Schoenfelder PG, et al. Chronic Obstructive Pulmonary Disease: A Community-Oriented Program Including Professional Education and Screening by a Voluntary Health Agency. :8.
39. Jordan RE, Lam K b. H, Cheng KK, Miller MR, Marsh JL, Ayres JG, et al. Case finding for chronic obstructive pulmonary disease: a model for optimising a targeted approach. Thorax. 2010 Jun 1;65(6):492–8.
40. Iyer S, Rai SP, Singhania S, Simon C. Role of Screening Lung Function Tests in a Routine Health Checkup. Cureus. 2023 Nov;15(11):e49430.
41. Korczyński P, Górska K, Jankowski P, Kosiński J, Kudas A, Sułek K, et al. Public spirometry campaign in chronic obstructive pulmonary disease screening - hope or hype? Adv Respir Med. 2017;85(3):143–50.
42. Lambert AA, Drummond MB, Kisalu A, Moxley J, Keruly J, Moore RD, et al. Implementation of a COPD Screening Questionnaire in an Outpatient HIV Clinic. COPD. 2016 Dec;13(6):767–72.
43. Lyngsø AM, Gottlieb V, Backer V, Nybo B, Østergaard MS, Jørgensen HL, et al. Early Detection of COPD in Primary Care: The Copenhagen COPD Screening Project. COPD J Chronic Obstr Pulm Dis. 2013 Mar 21;10(2):208–15.
44. Maio S, Sherrill DL, MacNee W, Lange P, Costabel U, Dahlén SE, et al. The European Respiratory Society spirometry tent: a unique form of screening for airway obstruction. Eur Respir J. 2012 Jun;39(6):1458–67.

45. Manzar N, Haque AS, Manzar B, Irfan M. The Efficacy of Spirometry as a Screening Tool in Detection of Air Flow Obstruction. Open Respir Med J. 2010 Sep 23;4:71–5.
46. Oike T, Senjyu H, Higa N, Kozu R, Tanaka T, Asai M, et al. Detection of Airflow Limitation Using the 11-Q and Pulmonary Function Tests. Intern Med. 2013;52(8):887–93.
47. Qazi HA, Soomro JA, Soomro TK, Soomro FA, Rasheed F, Hashmi A. Spirometric Screening of Chronic Obstructive Pulmonary Disease in Smokers Presenting to Tertiary Care Centre. J Med. 2009;10(2):40–4.
48. Riegels-Jakobsen T, Skouboe M, Dollerup J, Andersen CB, Staal LB, Jakobsen RBH, et al. Municipality screening of citizens with suspicion of chronic obstructive pulmonary disease. Int J Chron Obstruct Pulmon Dis. 2012;7:35–41.
49. Robitaille C, Dajczman E, Hirsch AM, Small D, Ernst P, Porubska D, et al. Implementation of a Targeted Screening Program to Detect Airflow Obstruction Suggestive of Chronic Obstructive Pulmonary Disease within a Presurgical Screening Clinic. Can Respir J. 2015;22(4):209–14.
50. Sekine Y, Yanagibori R, Suzuki K, Sugiyama S, Yamaji H, Ishibashi M, et al. Surveillance of chronic obstructive pulmonary disease in high-risk individuals by using regional lung cancer mass screening. Int J Chron Obstruct Pulmon Dis. 2014 Jun 23;9(1):647–56.
51. Sekine Y, Fujisawa T, Suzuki K, Tsutatani S, Kubota K, Ikegami H, et al. Detection of chronic obstructive pulmonary disease in community-based annual lung cancer screening: Chiba Chronic Obstructive Pulmonary Disease Lung Cancer Screening Study Group. Respirology. 2014;19(1):98–104.
52. Tabori D, Acketa M, Goldman S, Beljanski-Conkić R, Conkić B, Todić V, et al. Mass screening for early detection of bronchial obstruction. Its possibilities and benefits? Bronchopneumologie. 1980;30(6):471–84.
53. Ulrik CS, Løkke A, Dahl R, Dollerup J, Hansen G, Cording PH, et al. Early detection of COPD in general practice. Int J Chron Obstruct Pulmon Dis. 2011;6:123–7.
54. Schayck CP van, Loozen JMC, Wagena E, Akkermans RP, Wesseling GJ. Detecting patients at a high risk of developing chronic obstructive pulmonary disease in general practice: cross sectional case finding study. BMJ. 2002 Jun 8;324(7350):1370.
55. Vandevoorde J, Verbanck S, Gijssels L, Schuermans D, Devroey D, De Backer J, et al. Early detection of COPD: A case finding study in general practice. Respir Med. 2007 Mar;101(3):525–30.
56. Wang S, Gong W, Tian Y. Voluntary pulmonary function screening identifies high rates of undiagnosed asymptomatic chronic obstructive pulmonary disease. Chron Respir Dis. 2016 May;13(2):137–43.
57. Wisnivesky J, Skloot G, Rundle A, Revenson TA, Neugut A. Spirometry screening for airway obstruction in asymptomatic smokers. Aust Fam Physician. 2014 Jul;43(7):463–7.
58. Rokach A, Bohadana A, Kotek O, Shuali CC, Azulai H, Babai P, et al. Early Detection of COPD: An Opportunistic Case Finding Study in Smokers and Ex-Smokers Visiting a Medical Centre. Int J Chron Obstruct Pulmon Dis. 2021 Jun 1;16:1519–27.
59. Shirley DK, Kaner RJ, Glesby MJ. Screening for Chronic Obstructive Pulmonary Disease (COPD) in an Urban HIV Clinic: A Pilot Study. AIDS Patient Care STDs. 2015 May 1;29(5):232–9.
60. Soriano JB, Molina J, Miravitlles M. Combining case-finding methods for COPD in primary care: a large, two-stage design study. Int J Tuberc Lung Dis. 2018 Jan 1;22(1):106–11.

61. Mahboub B, Alzaabi A, Soriano JB, Salameh L, Mutairi YA, Yusufali AA, et al. Case-finding of chronic obstructive pulmonary disease with questionnaire, peak flow measurements and spirometry: a cross-sectional study. BMC Res Notes. 2014 Apr 16;7(1):241.
62. Al Ghobain M, Al-Hajjaj MS, Wali SO. Prevalence of chronic obstructive pulmonary disease among smokers attending primary healthcare clinics in Saudi Arabia. Ann Saudi Med. 2011;31(2):129–33.
63. Aaron SD, Vandemheen KL, Whitmore GA, Bergeron C, Boulet LP, Côté A, et al. Early Diagnosis and Treatment of COPD and Asthma — A Randomized, Controlled Trial. New England Journal of Medicine. 2024 Jun 12;390(22):2061–73.
64. Alhabeeb FF, Whitmore GA, Vandemheen KL, FitzGerald JM, Bergeron C, Lemière C, et al. Disease burden in individuals with symptomatic undiagnosed asthma or COPD. Respir Med [Internet]. 2022 Aug 1 [cited 2023 Jun 29];200. Available from: https://www.resmedjournal.com/article/S0954-6111(22)00182-2/fulltext
65. Andreeva E, Pokhaznikova M, Lebedev A, Moiseeva I, Kutznetsova O, Degryse JM. The Prevalence of Chronic Obstructive Pulmonary Disease by the Global Lung Initiative Equations in North-Western Russia. Respiration. 2016 Jan 5;91(1):43–55.
66. Bahtouee M, Maleki N, Nekouee F. The prevalence of chronic obstructive pulmonary disease in hookah smokers. Chron Respir Dis. 2018 May;15(2):165–72.
67. Bertens LCM, Reitsma JB, van Mourik Y, Lammers JWJ, Moons KGM, Hoes AW, et al. COPD detected with screening: impact on patient management and prognosis. Eur Respir J. 2014 Dec;44(6):1571–8.
68. Bui DS, Burgess JA, Lowe AJ, Perret JL, Lodge CJ, Bui M, et al. Childhood Lung Function Predicts Adult Chronic Obstructive Pulmonary Disease and Asthma–Chronic Obstructive Pulmonary Disease Overlap Syndrome. Am J Respir Crit Care Med. 2017 Jul;196(1):39–46.
69. Bunker J, Hermiz O, Zwar N, Dennis SM, Vagholkar S, Crockett A, et al. Feasibility and efficacy of COPD case finding by practice nurses. Aust Fam Physician. 2009 Oct;38(10):826–30.
70. Dirven JAM, Muris JWM, van Schayck CP. COPD Screening in General Practice Using a Telephone Questionnaire. COPD J Chronic Obstr Pulm Dis. 2010 Sep 1;7(5):352–9.
71. Chapron A, Andres E, Fiquet L, Pelé F, Allory E, Le Pabic E, et al. Early detection of chronic obstructive pulmonary disease in primary care: a randomised controlled trial. Br J Gen Pract. 2023 Dec;73(737):e876–84.
72. Grzetic- Romcevic T, Devcic B. Spirometric testing on World COPD Day. Int J Chron Obstruct Pulmon Dis. 2011 Feb;141.
73. Hvidsten SC, Storesund L, Wentzel-Larsen T, Gulsvik A, Lehmann S. Prevalence and predictors of undiagnosed chronic obstructive pulmonary disease in a Norwegian adult general population. Clin Respir J. 2010 Jan;4(1):13–21.
74. Hanibuchi M, Saijo A, Mitsuhashi A, Kajimoto T, Kitagawa T, Nishioka Y. The efficacy of mass screening for chronic obstructive pulmonary disease using screening questionnaires in a medical health check-up population. Respiratory Investigation. 2022 Nov;60(6):815–21.
75. Jithoo A, Enright P, Burney P, Buist AS, Bateman ED, Tan WC, et al. Case-finding options for COPD: Results from the BOLD Study. Eur Respir J. 2013 Mar;41(3):548–55.
76. Johnson KM, Tan WC, Bourbeau J, Sin DD, Sadatsafavi M, Bourbeau J, et al. The diagnostic performance of patient symptoms in screening for COPD. Respir Res. 2018 Aug 3;19(1):147.

77. Jordan RE, Adab P, Sitch A, Enocson A, Blissett D, Jowett S, et al. Targeted case finding for chronic obstructive pulmonary disease versus routine practice in primary care (TargetCOPD): a cluster-randomised controlled trial. Lancet Respir Med. 2016 Sep 1;4(9):720–30.
78. KalagoudaMahishale V, Angadi N, Metgudmath V, Lolly M, Eti A, Khan S. The Prevalence of Chronic Obstructive Pulmonary Disease and the Determinants of Underdiagnosis in Women Exposed to Biomass Fuel in India- a Cross Section Study. Chonnam Med J. 2016 May;52(2):117–22.
79. Kart L, Akkoyunlu ME, Bayram M, Yakar F, Kutbay Özçelik H, Karaköse F, et al. COPD: an underdiagnosed disease at hospital environment. Wien Klin Wochenschr. 2014 Feb;126(3–4):73–8.
80. Ulrik C, Kjeldgaard P, Dahl R, Løkke A. Detection of COPD in a high-risk population: should the diagnostic work-up include bronchodilator reversibility testing? Int J Chron Obstruct Pulmon Dis. 2015 Feb;407.
81. Kögler H, Metzdorf N, Glaab T, Welte T. Preselection of patients at risk for COPD by two simple screening questions. Respir Med. 2010 Jul;104(7):1012–9.
82. Laniado-Laborin R, Rendón A, Bauerle O. Chronic obstructive pulmonary disease case finding in Mexico in an at-risk population. :7.
83. Lewis-Burke N, Vlies B, Wooding O, Davies L, Walker PP. A Screening Study to Determine the Prevalence of Airway Disease in Heroin Smokers. COPD J Chronic Obstr Pulm Dis. 2016 May 3;13(3):333–8.
84. López Varela MV, Montes de Oca M, Rey A, Casas A, Stirbulov R, Di Boscio V, et al. Development of a simple screening tool for opportunistic COPD case finding in primary care in Latin America: The PUMA study: COPD case finding in primary care. Respirology. 2016 Oct;21(7):1227–34.
85. Mahishale V, Mahishale A, Angadi N, Metgudmath V, Eti A, Lolly M, et al. Screening for chronic obstructive pulmonary disease in elderly subjects with dyspnoea and/or reduced exercise tolerance – A hospital based cross sectional study. Egypt J Chest Dis Tuberc. 2015 Jul 1;64(3):567–71.
86. Midtgarden JM, Renstrøm N, Obling N, Bodtger U. Screening for obstructive lung disease in hospitalized psychiatric patients. Nord J Psychiatry. 2020 Apr 2;74(3):181–6.
87. Nascimento OA, Camelier A, Rosa FW, Menezes AMB, Pérez-Padilla R, Jardim JR, et al. Chronic obstructive pulmonary disease is underdiagnosed and undertreated in São Paulo (Brazil): results of the PLATINO study. Braz J Med Biol Res Rev Bras Pesqui Medicas E Biol. 2007 Jul;40(7):887–95.
88. Preteroti M, Whitmore GA, Vandemheen KL, FitzGerald JM, Lemière C, Boulet LP, et al. Population-based case-finding to identify subjects with undiagnosed asthma or COPD. Eur Respir J [Internet]. 2020 Jun 1 [cited 2023 Jun 29];55(6). Available from: https://erj.ersjournals.com/content/55/6/2000024
89. Pagano L, Dennis S, Wootton S, Mahadev S, Chan ASL, Zwar N, et al. Identifying airway obstruction in primary care: is there a role for physiotherapists? BMC Prim Care. 2022 Dec 14;23(1):324.
90. Sansores RH, Ramírez-Venegas A, Hernández-Zenteno R, Mayar-Maya ME, Pérez-Bautista OG, Velázquez Uncal M. Prevalence and diagnosis of chronic obstructive pulmonary disease among smokers at risk. A comparative study of case-finding vs. screening strategies. Respir Med. 2013 Apr 1;107(4):580–6.
91. Sang L, Gong X, Huang Y, Sun J. Proportions and risk factors of chronic obstructive pulmonary disease and preserved ratio impaired spirometry, and association with small airway disease, in the positive screening older population from China: a cross-sectional study. BMC Pulm Med. 2024 Mar 5;24(1):114.

92. Schirnhofer L, Lamprecht B, Firlei N, Kaiser B, Buist AS, Halbert RJ, et al. Using Targeted Spirometry to Reduce Non-Diagnosed Chronic Obstructive Pulmonary Disease. Respiration. 2010 Aug 19;81(6):476–82.
93. Siddharthan T, Pollard SL, Quaderi SA, Rykiel NA, Wosu AC, Alupo P, et al. Discriminative Accuracy of Chronic Obstructive Pulmonary Disease Screening Instruments in 3 Low- and Middle-Income Country Settings. JAMA. 2022 Jan 11;327(2):151–60.
94. Sinha B, Vibha, Singla R, Chowdhury R. An epidemiological profile of chronic obstructive pulmonary disease: A community-based study in Delhi. J Postgrad Med. 2017;63(1):29.
95. Skucha W, Mejza F, Nastalek P, Doniec Z. Pulmonary prevention program in the Proszowice county: description and results. Adv Respir Med. 2017;85(5):239–45.
96. Stav D, Raz M. Prevalence of chronic obstructive pulmonary disease among smokers aged 45 and up in Israel. Isr Med Assoc J IMAJ. 2007 Nov;9(11):800–2.
97. Su KC, Ko HK, Chou KT, Hsiao YH, Su VYF, Perng DW, et al. An accurate prediction model to identify undiagnosed at-risk patients with COPD: a cross-sectional case-finding study. Npj Prim Care Respir Med. 2019 May 28;29(1):1–7.
98. van Mourik Y, Bertens LCM, Cramer MJM, Lammers JWJ, Reitsma JB, Moons KGM, et al. Unrecognized heart failure and chronic obstructive pulmonary disease (COPD) in frail elderly detected through a near-home targeted screening strategy. J Am Board Fam Med JABFM. 2014;27(6):811–21.
99. Tang W, Rong Y, Zhang H, Lin W, Zeng W, Wu W. Screening and early diagnosis of chronic obstructive pulmonary disease: a population study. BMC Pulm Med. 2023 Nov 3;23:424.
100. van Mourik Y, Rutten FH, Bertens LCM, Cramer MJM, Lammers JWJ, Gohar A, et al. Clinical research study implementation of case-finding strategies for heart failure and chronic obstructive pulmonary disease in the elderly with reduced exercise tolerance or dyspnea: A cluster randomized trial. Am Heart J. 2020 Feb;220:73–81.
101. Zachariades AG, Zachariadou T, Adamide T, Anagnostopoulou U, Georgiou A, Gourgoulianis KI. Prevalence of Chronic Obstructive Pulmonary Disease in Cyprus: A Population-Based Study. COPD J Chronic Obstr Pulm Dis. 2012 May 23;9(3):259–67.
102. Yang S, Yin X, Zhang Y, Zhao H, Zheng Z, Li J, et al. Efficacy of a Self-Designed Questionnaire for Community Screening of COPD. Int J Chron Obstruct Pulmon Dis. 2022 Jun 14;17:1381–91.
103. Zubair T, Abbassi A, Khan OA. Early Detection of Chronic Obstructive Pulmonary Disease in Apparently Healthy Attendants of Tertiary Care Hospital and Assessment of its Severity. J Coll Physicians Surg--Pak JCPSP. 2017 May;27(5):296–300.
104. Agarwal D, Hanafi NS, Khoo EM, Parker RA, Ghorpade D, Salvi S, et al. Predictors for detecting chronic respiratory diseases in community surveys: A pilot cross-sectional survey in four South and South East Asian low- and middle-income countries. J Glob Health. 11:04065.
105. ALAM DS, CHOWDHURY MA, SIDDIQUEE AT, AHMED S, CLEMENS JD. Prevalence and determinants of Chronic Obstructive Pulmonary Disease (COPD) in Bangladesh. COPD. 2015;12(6):658–67.
106. Bednarek M, Maciejewski J, Wozniak M, Kuca P, Zielinski J. Prevalence, severity and underdiagnosis of COPD in the primary care setting. Thorax. 2008 May 1;63(5):402–7.

107. Burhan H, Young R, Byrne T, Peat R, Furlong J, Renwick S, et al. Screening Heroin Smokers Attending Community Drug Services for COPD. Chest. 2019 Feb;155(2):279–87.
108. Demirci H, Eniste K, Basaran EO, Ocakoglu G, Yilmaz Z, Tuna S. A multicenter family practitioners' research on Chronic Obstructive Pulmonary Disease screening using the COPD Assessment Test. Prim Health Care Res Dev. 2017 Nov;18(6):603–7.
109. Dickinson JA, Meaker M, Searle M, Ratcliffe G. Screening older patients for obstructive airways disease in a semi-rural practice. Thorax. 1999 Jun 1;54(6):501–5.
110. Geijer RM, Sachs AP, Hoes AW, Salomé PL, Lammers JWJ, Verheij TJ. Prevalence of undetected persistent airflow obstruction in male smokers 40–65 years old. Fam Pract. 2005 Oct 1;22(5):485–9.
111. Geijer RMM, Sachs APE, Verheij TJM, Lammers JWJ, Salomé PL, Hoes AW. Are patient characteristics helpful in recognizing mild COPD (GOLD I) in daily practice? Scand J Prim Health Care. 2006 Jan 1;24(4):237–42.
112. Gu Y, Zhang Y, Wen Q, Ouyang Y, Shen Y, Yu H, et al. Performance of COPD population screener questionnaire in COPD screening: a validation study and meta-analysis. Ann Med. 2021 Jan 1;53(1):1199–207.
113. Hagstad S, Ekerljung L, Lindberg A, Backman H, Rönmark E, Lundbäck B. COPD among non-smokers – Report from the Obstructive Lung Disease in Northern Sweden (OLIN) studies. Respir Med. 2012 Jul;106(7):980–8.
114. Hill K, Goldstein RS, Guyatt GH, Blouin M, Tan WC, Davis LL, et al. Prevalence and underdiagnosis of chronic obstructive pulmonary disease among patients at risk in primary care. Can Med Assoc J. 2010 Apr 20;182(7):673–8.
115. Jan S, Metten MA, Chapron A, Marette S, Robert AM, Guillot S, et al. Use of the COPD Assessment Test (CAT) to screen for COPD in dairy farmers: AIRBAg study. Clin Respir J. 2020;14(9):813–21.
116. Khassawneh BY, Samrah SM, Jarrah MI, Ibdah RK, Ibnian AM, Almistarehi AW, et al. Prevalence of undiagnosed COPD in male patients with coronary artery disease: a cross-sectional study in Jordan. Int J Chron Obstruct Pulmon Dis. 2018 Sep;Volume 13:2759–66.
117. Konstantikaki V, Kostikas K, Minas M, Batavanis G, Daniil Z, Gourgoulianis KI, et al. Comparison of a network of primary care physicians and an open spirometry programme for COPD diagnosis. Respir Med. 2011 Feb;105(2):274–81.
118. Kotaki K, Ikeda H, Fukuda T, Yuki F, Hasuo K, Kawano Y, et al. Effectiveness of diagnostic screening tests in mass screening for COPD using a cooperative regional system in a region with heavy air pollution: a cross-sectional study. BMJ Open. 2017 Jan;7(1):e012923.
119. Laraqui O, Hammouda R, Laraqui S, Manar N, Ghailan T, Ben Amor J, et al. Prevalence of chronic obstructive respiratory diseases amongst fishermen. Int Marit Health. 2018;69(1):13–21.
120. Llordés M, Jaén A, Almagro P, Heredia JL, Morera J, Soriano JB, et al. Prevalence, Risk Factors and Diagnostic Accuracy of COPD Among Smokers in Primary Care. COPD J Chronic Obstr Pulm Dis. 2015 Jul 4;12(4):404–12.
121. Løkke A, Ulrik CS, Dahl R, Plauborg L, Dollerup J, Kristiansen LC, et al. Detection of previously undiagnosed cases of COPD in a high-risk population identified in general practice. COPD. 2012 Aug;9(5):458–65.
122. Lu M, Yao W, Zhong N, Zhou Y, Wang C, Chen P, et al. Chronic obstructive pulmonary disease in the absence of chronic bronchitis in China: COPD without chronic bronchitis. Respirology. 2010 Aug 16;15(7):1072–8.

123. Mycroft K, Korczynski P, Jankowski P, Kutka M, Zelazna O, Zagaja M, et al. Active screening for COPD among hospitalized smokers – a feasibility study. Ther Adv Chronic Dis. 2020 Jan 1;11:2040622320971111.
124. Martinez FJ, Han MK, Lopez C, Murray S, Mannino D, Anderson S, et al. Discriminative Accuracy of the CAPTURE Tool for Identifying Chronic Obstructive Pulmonary Disease in US Primary Care Settings. JAMA. 2023 Feb 14;329(6):490.
125. Ozemek C, Arena R, Rouleau CR, Campbell TS, Hauer T, Wilton SB, et al. Identification of Patients With COPD in a Cardiac Rehabilitation Setting : THE COnCuR STUDY. J Cardiopulm Rehabil Prev. 2021 May 1;41(3):172–5.
126. Quiros-Roldan E, Pezzoli MC, Berlendis M, Raffetti E, Ferraresi A, Properzi M, et al. A COPD Case-Finding Program in a Large Cohort of HIV-Infected Persons. Respir Care. 2019 Feb 1;64(2):169–75.
127. Spyratos D, Chloros D, Haidich AB, Hatzidimitriou N, Karoulias A, Nella D, et al. Comparison among three screening questionnaires for COPD diagnosis in the primary care. Eur Respir J [Internet]. 2013 Sep 1 [cited 2023 Jun 30];42(Suppl 57). Available from: https://erj.ersjournals.com/content/42/Suppl_57/P266
128. Stafyla E, Kotsiou OS, Deskata K, Gourgoulianis K. Missed diagnosis and overtreatment of COPD among smoking primary care population in Central Greece: old problems persist. Int J Chron Obstruct Pulmon Dis. 2018 Feb;Volume 13:487–98.
129. Stanley AJ, Hasan I, Crockett AJ, van Schayck OCP, Zwar NA. COPD Diagnostic Questionnaire (CDQ) for selecting at-risk patients for spirometry: a cross-sectional study in Australian general practice. Npj Prim Care Respir Med. 2014 Nov;24(1):14024.
130. Stratelis G, Jakobsson P, Molstad S, Zetterstrom O. Early detection of COPD in primary care: screening by invitation of smokers aged 40 to 55 years. Br J Gen Pract. 2004;6.
131. Undrunas A, Kasprzyk P, Rajca A, Kuziemski K, Rzyman W, Zdrojewski T. Prevalence, symptom burden and under-diagnosis of chronic obstructive pulmonary disease in Polish lung cancer screening population: a cohort observational study. BMJ Open. 2022 Apr 1;12(4):e055007.
132. Zhou J, Yu N, Li X, Wang W. Accuracy of Six Chronic Obstructive Pulmonary Disease Screening Questionnaires in the Chinese Population. Int J Chron Obstruct Pulmon Dis. 2022 Feb 10;17:317–27.
133. Zhao X, Kang H, An Y, Xu Z, Wei M, Zhang Q, et al. Whole-course management of chronic obstructive pulmonary disease in primary healthcare: an internet of things-enabled prospective cohort study in China. BMJ Open Resp Res. 2024 Apr;11(1):e001954.
134. Abbas AH, Khadim HW, Jasim AH, Al-Hindy HAAM, Hammoud SS. Early detection and diagnosis of chronic obstructive pulmonary disease in asymptomatic male smokers and ex-smokers using spirometry. 2021 Jul 15 [cited 2023 Jun 29]; Available from: https://zenodo.org/record/5106415
135. Capozzolo A, Castellana G, Dragonieri S, Carratù P, Liotino V, Vulpi MR, et al. Voluntary lung function screening to reveal new COPD cases in southern Italy. Int J Chron Obstruct Pulmon Dis. 2017 Jul;Volume 12:2035–42.
136. Dirven JA, Tange HJ, Muris JW, van Haaren KM, Vink G, van Schayck OC. Early detection of COPD in general practice: implementation, workload and socioeconomic status. A mixed methods observational study. Prim Care Respir J J Gen Pract Airw Group. 2013 Sep;22(3):338–43.
137. Hanibuchi M, Saijo A, Mitsuhashi A, Kajimoto T, Kitagawa T, Nishioka Y. The efficacy of mass screening for chronic obstructive pulmonary disease using screening questionnaires in a medical health check-up population. Respir Investig. 2022 Nov 1;60(6):815–21.

138. Lee L, Patel T, Hillier LM, Milligan J. Office-Based Case Finding for Chronic Obstructive Pulmonary Disease in Older Adults in Primary Care. Can Respir J. 2016;2016:1–7.
139. Ray E, Culliford D, Kruk H, Gillett K, North M, Astles CM, et al. Specialist respiratory outreach: a case-finding initiative for identifying undiagnosed COPD in primary care. Npj Prim Care Respir Med. 2021 Feb 11;31(1):1–8.
140. Toljamo T, Kaukonen M, Nieminen P, Kinnula VL. Early detection of COPD combined with individualized counselling for smoking cessation: A two-year prospective study. Scand J Prim Health Care. 2010 Jan;28(1):41–6.
141. Tsukuya G, Matsumoto K, Fukuyama S, Crawford B, Nakanishi Y, Ichinose M, et al. Validation of a COPD screening questionnaire and establishment of diagnostic cut-points in a Japanese general population: The Hisayama study. Allergol Int. 2015 Jan;64(1):49–53.
142. Yang S, Yin X, Zhang Y, Zhao H, Zheng Z, Li J, et al. Efficacy of a Self-Designed Questionnaire for Community Screening of COPD. Int J Chron Obstruct Pulmon Dis. 2022 Jun 14;17:1381–91.
143. Kjeldgaard P, Lykkegaard J, Spillemose H, Ulrik CS. Multicenter study of the COPD-6 screening device: feasible for early detection of chronic obstructive pulmonary disease in primary care? Int J Chron Obstruct Pulmon Dis. 2017 Aug 4;12:2323–31.
144. Represas-Represas C, Fernández-Villar A, Ruano-Raviña A, Priegue-Carrera A, Botana-Rial M, study group of "Validity of COPD-6 in non-specialized healthcare settings." Screening for Chronic Obstructive Pulmonary Disease: Validity and Reliability of a Portable Device in Non-Specialized Healthcare Settings. Chotirmall SH, editor. PLOS ONE. 2016 Jan 4;11(1):e0145571.
145. Labor M, Vrbica Ž, Gudelj I, Labor S, Plavec D. Diagnostic accuracy of a pocket screening spirometer in diagnosing chronic obstructive pulmonary disease in general practice: a cross sectional validation study using tertiary care as a reference. BMC Fam Pract. 2016 Dec;17(1):112.
146. Alchakaki A, Riehani A, Shikh-Hamdon M, Mina N, Badr MS, Sankari A. Expiratory Snoring Predicts Obstructive Pulmonary Disease in Patients with Sleep-disordered Breathing. Ann Am Thorac Soc. 2016 Jan;13(1):86–92.
147. Catlett G, Kidera G. Detection of respiratory impairment in pilots. Aerosp Med. 1969;40(11):1252–7.
148. Cristescu SM, Gietema HA, Blanchet L, Kruitwagen CLJJ, Munnik P, van Klaveren RJ, et al. Screening for emphysema via exhaled volatile organic compounds. J Breath Res. 2011 Dec 1;5(4):046009.
149. Fidalgo-Garrido JW, Martinez-Carrasco JL. Early Diagnosis of Pulmonary Emphysema in Smokers. Ann N Y Acad Sci. 1991 May;624(1):362–4.
150. Markun S, Rosemann T, Dalla-Lana K, Steurer-Stey C. The Impact of Case Finding on the Recruitment Yield for COPD Research in Primary Care: An Observational Study. Respiration. 2016;92(5):308–15.
151. Mets OM, Buckens CFM, Zanen P, Isgum I, van Ginneken B, Prokop M, et al. Identification of chronic obstructive pulmonary disease in lung cancer screening computed tomographic scans. JAMA. 2011 Oct 26;306(16):1775–81.
152. Takahashi T, Ichinose M, Inoue H, Shirato K, Hattori T, Takishima T. Underdiagnosis and undertreatment of COPD in primary care settings. Respirology. 2003 Dec;8(4):504–8.
153. Boschetto P, Fucili A, Stendardo M, Malagù M, Parrinello G, Casimirri E, et al. Occurrence and impact of chronic obstructive pulmonary disease in elderly patients with stable heart failure. Respirology. 2013;18(1):125–30.
154. Brenner S, Güder G, Berliner D, Deubner N, Fröhlich K, Ertl G, et al. Airway obstruction in systolic heart failure – COPD or congestion? Int J Cardiol. 2013 Oct;168(3):1910–6.

155. Hamers R, Bontemps S, van den Akker M, Souza R, Penaforte J, Chavannes N. Chronic obstructive pulmonary disease in Brazilian primary care: diagnostic competence and case-finding. Prim Care Respir J. 2006 Oct 1;15(5):299–306.
156. Mosharraf-Hossain KM, Islam S, Kalam Azzad A, Pasha MM, Sultana F, Hossain RZ, et al. Detection of chronic obstructive pulmonary disease using spirometric screening. Mymensingh Med J MMJ. 2009 Jan;18(1 Suppl):S108-112.
157. Queiroz MC de CAM de, Moreira MAC, Rabahi MF. Subdiagnóstico de DPOC na atenção primária em Aparecida de Goiânia, Goiás. J Bras Pneumol. 2012 Dec;38(6):692–9.
158. Steinacher R, Parissis JT, Strohmer B, Eichinger J, Rottlaender D, Hoppe UC, et al. Comparison between ATS/ERS age- and gender-adjusted criteria and GOLD criteria for the detection of irreversible airway obstruction in chronic heart failure. Clin Res Cardiol. 2012 Aug;101(8):637–45.
159. Clergue-Duval V, Lair R, Lefebvre-Durel C, Barré T, Gautron MA, Mehtelli W, et al. COPD Positive Screening with Spirometry Increases Motivation to Quit Tobacco Smoking in an Addiction Treatment Center. COPD J Chronic Obstr Pulm Dis. 2020 May 3;17(3):240–4.
160. Freeman D, Nordyke RJ, Isonaka S, Nonikov DV, Maroni JM, Price D, et al. Questions for COPD diagnostic screening in a primary care setting. Respir Med. 2005 Oct;99(10):1311–8.
161. Mooe T, Stenfors N. The Prevalence of COPD in Individuals with Acute Coronary Syndrome: A Spirometry-Based Screening Study. COPD J Chronic Obstr Pulm Dis. 2015 Jul 4;12(4):453–61.
162. Nathell L, Nathell M, Malmberg P, Larsson K. COPD diagnosis related to different guidelines and spirometry techniques. Respir Res. 2007 Dec;8(1):89.
163. Ronaldson SJ, Dyson L, Clark L, Hewitt CE, Torgerson DJ, Cooper BG, et al. Determining the optimal approach to identifying individuals with chronic obstructive pulmonary disease: The DOC study. J Eval Clin Pract. 2018;24(3):487–95.
164. Sandelowsky H, Ställberg B, Nager A, Hasselström J. The prevalence of undiagnosed chronic obstructive pulmonary disease in a primary care population with respiratory tract infections - a case finding study. BMC Fam Pract. 2011 Dec;12(1):122.
165. Tupper OD, Kjeldgaard P, Løkke A, Ulrik CS. Predictors of COPD in symptomatic smokers and ex-smokers seen in primary care. Chron Respir Dis. 2018 Nov;15(4):393–9.
166. Vrijhoef HJM, Diederiks JPM, Wesseling GJ, Van Schayck CP, Spreeuwenberg C. Undiagnosed patients and patients at risk for COPD in primary health care: early detection with the support of non-physicians: Early detection of COPD with support of non-physicians. J Clin Nurs. 2003 May;12(3):366–73.
167. Apostolovic S, Jankovic-Tomasevic R, Salinger-Martinovic S, Djordjevic-Radojkovic D, Stanojevic D, Pavlovic M, et al. Frequency and significance of unrecognized chronic obstructive pulmonary disease in elderly patients with stable heart failure. Aging Clin Exp Res. 2011 Oct 1;23(5):337–42.
168. Barthwal M, Singh S. Early Detection of Chronic Obstructive Pulmonary Disease in Asymptomatic Smokers using Spirometry. 2014 Mar;62(ORIGINAL ARTICLE). Available from: https://www.japi.org/r2c474c4/early-detection-of-chronic-obstructive-pulmonary-disease-in-asymptomatic-smokers-using-spirometry#references
169. Marcos PJ, Malo de Molina R, Casamor R. Risk stratification for COPD diagnosis through an active search strategy in primary care. Int J Chron Obstruct Pulmon Dis. 2016 Mar 1;11:431–7.

170. Tinkelman DG, Price DB, Nordyke RJ, Halbert RJ. COPD screening efforts in primary care: what is the yield? Prim Care Respir J J Gen Pract Airw Group. 2007 Feb;16(1):41–8.

Makhinova T, et al. Informing community pharmacists on COPD case-finding methods: a scoping review. *Can Pharm J* (Ott) 2024;157. DOI: 10.1177/17151635231284802.
